# Recovery capital and substance(s) of choice among adolescents: Exploratory analyses

**DOI:** 10.1016/j.abrep.2026.100759

**Published:** 2026-10-13

**Authors:** Jules Boyle, Jordan Jurinsky

**Affiliations:** Eliot-Pearson Department of Child Study and Human Development, Tufts University, 105 College Ave, Medford, MA, 02155, United States of America

## Abstract

**Introduction:**

Recovery capital is the availability of resources to support recovery. Prior research suggests a relationship between substances of choice (SOC) and recovery capital. This study explored the relationship between SOC and recovery capital among adolescents attending a recovery high school.

**Methods:**

A secondary data analysis of data from the SEARS project was conducted. Participants (*N* = 624, age_mean_ = 16.36) completed a modified Assessment of Recovery Capital (ARC) and responded to the question: “What is your drug of choice? (Please select all that apply)”. Substances were grouped into type of SOC (alcohol, cannabis, opioids, stimulants, or other) and number of SOC (one, two, three or more, or “whatever is available”).

**Results:**

A Kruskal-Wallis test to compare recovery capital across number of SOC groups indicated a significant difference, χ2(*df* = 3, *N* = 624) = 8.17, *p* = .043. Post hoc comparisons using Dunn's test identified that participants who selected “whatever is available” reported lower recovery capital than participants who preferred one SOC, *p*_*adjusted*_ = 0.022. There were no significant differences in recovery capital across type of SOC.

**Discussion:**

The current study identified a relationship between number of SOC and recovery capital, suggesting those who prefer “whatever is available” may benefit from additional support. More work is needed to assess the utility and implications of SOC among adolescents in recovery. Researchers who ask participants about SOC may consider allowing participants to select multiple substances and including an option for “whatever is available” to better capture use preferences.

## Highlights

• Number of substances of choice was related to recovery capital among adolescents.
• Preference of “whatever is available” was linked to lower recovery capital.
• There were some differences between personal and social recovery capital.
• Preferences rather than use patterns may have measurement utility.
• Researchers may consider inclusion of broader preferences in survey design.

## 1 Introduction

In 2024, approximately 2,024,000 adolescents (aged 12–17) in the U.S. met criteria for a substance use disorder (SUD; Substance Abuse and Mental Health Services Administration, 2024). Interest in and opportunities for substance use often arise during adolescence (Jackson, 2019). Initiating use during adolescence is associated with continued use, polysubstance use, and greater odds of developing a SUD in adulthood (Kelly et al., 2019; Millar et al., 2021; Volkow et al., 2021). Importantly, many adolescents report resolving their substance use problems, and some identify as being in recovery (Smith et al., 2023). Adolescents pursue recovery through multiple pathways and understanding relevant resources can inform the development and delivery of recovery services (Finch et al., 2020).

### 1.1 Recovery capital (RC) and recovery high schools (RHSs)

RC refers to the availability of internal and external resources to support recovery (Cloud & Granfield, 2008). It is a strengths-based ecological model which includes individual, inter-individual, and environmental-level factors. RC can influence an individual's recovery progression, with greater RC supporting better recovery outcomes (Jurinsky et al., 2025; Laudet & White, 2008).

Community supports such as recovery housing, peer support services, mutual aid organizations (e.g., 12-step groups), and RHSs can support RC among adolescents. (Ashford et al., 2019; Hennessy et al., 2025). RHSs are secondary schools that support students in recovery and meet requirements to award a secondary school diploma. RHSs offer various supports which can include group and individual counseling, family involvement, and referrals to external services alongside traditional coursework (Finch et al., 2014). Additionally, RHSs may provide opportunities for adolescents in recovery to form a pro-recovery peer network (Finch & Frieden, 2014; Hennessy et al., 2024; Murphy, 2025; Yule & Kelly, 2018).

### 1.2 Polysubstance use and substance of choice (SOC)

Polysubstance use is the combined use of two or more substances over a defined period of time (Shearer et al., 2022; Xu et al., 2025). Polysubstance use may function to enhance effects of a particular substance, self-medicate, cope with distress, reduce cravings, and/or respond to social contexts (Boileau-Falardeau et al., 2022; Bunting et al., 2024). If individuals can use multiple substances, they can also prefer multiple substances. Limited research has examined polysubstance preferences or multiple SOC.

A SOC is the substance an individual prefers to use or most commonly uses. SOC is used in place of “drug of choice (DOC)” as the term “drug” can be stigmatizing due to its ambiguity in meaning and associated implications (Recovery Research Institute, 2026). SOC is frequently used in practitioner vernacular and as a covariate in addiction research (e.g., Gold, 2025). Prior research on SOC proposed the self-medication hypothesis, which posits that an individual's SOC is determined by the psychopharmacological properties of a substance and what they are seeking relief from (Khantzian, 1985, Khantzian, 1997). Research has begun to examine the role of SOC in recovery and suggests there are distinct recovery challenges related to different substances (Gilligan et al., 2017; Neale et al., 2014; The Society for Adolescent Health and Medicine, 2021). Nevertheless, recovery capital across SOC remains understudied, especially among adolescents.

Everyone with a SUD may have a SOC, yet this has not been empirically explored. Historically, a lack of substance preference was deemed by the stigmatizing term “garbage head syndrome” (Byqvist, 1999; Cohen, 1981; Gardner, 1981). Its presence highlights that a lack of preference or equal preference of many substances may be worth exploring to better understand substance use and recovery needs. To our knowledge, a lack of preference or equal preference of many substances has not been empirically measured at this time.

### 1.3 Current study

Previous research has identified the need to assess differences in RC across SOC (Bowen & Hennessy, 2025). This exploratory study examines the relationship between SOC and RC among adolescents attending a RHS in the U.S. The present study analyzes (a) the relationship between type of SOC and RC and (b) the relationship between number of SOC and RC.

## 2 Methods

### 2.1 Participants and procedures

This study is a secondary data analysis of cross-sectional data collected through the Systematic Evaluation of Adolescent Recovery Services (SEARS) project. SEARS is a comprehensive tool about recovery experiences administered in RHSs across the nation, with methods previously published (Jurinsky et al., 2025). Our analytic sample included 624 students (*mean age* = 16.36, *SD* = 1.34) attending 26 RHSs across the U.S. who had completed SEARS at the time of this study (February 2022–June 2025; Table 1).

**Table 1:** Baseline demographics for analytic sample (*N* = 624).

Group	Total sample		One SOC subgroup	
	*n*	%	*n*	%
Total	624	100%	172	27.56%
Gender				
Male	285	46.79%	86	51.81%
Female	290	47.62%	74	44.58%
Self-Identify	34	5.58%	6	3.61%
Race				
Black	43	6.98%	20	11.90%
White	406	65.91%	90	53.57%
Multiracial	83	13.47%	22	13.10%
Other	84	13.64%	36	21.43%
Ethnicity				
Latine	161	26.88%	53	33.33%
Non-Latine	438	73.12%	106	66.67%
Parent Education				
High School or Less	157	37.92%	46	41.07%
Some College/Technical	57	13.77%	13	11.61%
Bachelor's Degree	98	23.67%	25	22.32%
Advanced Degree	102	24.64%	28	25.00%
Enrolled in RHS at least 90 days?				
Yes	293	50.09%	78	48.75%
No	292	49.91%	82	51.25%

### 2.2 Measures

#### 2.2.1 RC

RC was measured using a modified Assessment of Recovery Capital (ARC). The ARC is a 50-item measure of RC with ten domains that can be grouped into personal and social RC (Groshkova et al., 2013). The overall ARC score is calculated by totaling all scores, with a higher score indicating higher RC. Six ARC items were slightly modified (Appendix, Table 3). For example, “I have no problems with transportation” was modified to read, “I have no problems getting around.”

#### 2.2.2 SOC

Participants were asked “What is your drug of choice? (Please select all that apply)” and provided with sixteen options: alcohol, heroin, fentanyl, crack cocaine, cocaine powder, medication for ADHD, methamphetamines, cannabis, medication for opioid use disorder, prescription pain pill, benzodiazepines, pressed pills with unspecified contents, over the counter medication, hallucinogens, whatever is available, and other substance. Participants could select any number or combination of substances. Frequencies of SOC selections are reported in the Appendix, Table 4.

##### 2.2.2.1 Type of SOC

Type of SOC were grouped into five categories: alcohol, cannabis, stimulants (crack cocaine, cocaine powder, ADHD medication, and methamphetamines), opioids (heroin, fentanyl, and prescription pain pills), and other (medications for opioid use disorder, benzodiazepines, pressed pills with unspecified contents, over-the-counter medication, hallucinogens, and selection of “Other substance”). Descriptive data for type of SOC and recovery capital are reported in the Appendix, Tables 5–7.

##### 2.2.2.2 Number of SOC

Participants were grouped into four mutually exclusive groups based on the number of SOC selected: one, two, three or more, or “whatever is available.” Number of substances was determined by the number of specific substances selected, not by the number of substance categories selected. Any participant who selected “whatever is available” was only placed into that category. Descriptive data for number of SOC and recovery capital are reported in the Appendix, Tables 8–10.

### 2.3 Data analysis

Data were analyzed in R (R Core Team, 2025). Listwise deletion was used to handle missing data (286 cases deleted for missingness: 49 cases missing SOC only, 199 cases missing ARC only, 38 cases missing both). As a sensitivity analysis, we repeated the analyses using prorated ARC scores for participants completing at least 90% of ARC items (≤5 missing items). Prorated scores were calculated by multiplying each participant's proportion of endorsed ARC items by the total number of ARC items. The sensitivity analyses yielded substantively similar findings; therefore, results from the original complete-case analyses are presented. Due to violations of normality in the data, Kruskal-Wallis tests were conducted instead of ANOVAs. Dunn's tests were used for post hoc comparisons. Bonferroni corrections were used to account for higher likelihood of a Type I error. To compare RC across types of SOC, only participants who selected one SOC (*n* = 172) were included in this analysis to meet assumptions of independence of observations. To compare RC across number of SOC categories, the full sample (*N* = 624) was used.

## 3 Results

### 3.1 Are there differences in RC across SOC categories?

The Kruskal-Wallis test indicated there was not a significant difference in RC across types of SOC. Similar findings emerged for the personal and social RC subdomains (Table 2).

**Table 2:** Kruskal-Wallis results for type of SOC and number of SOC.

Outcome	Groups	*df*	χ2	*p*
ARC (Sum)	Alcohol Cannabis Stimulant Opioid Other	4	6.98	0.137
ARC (Personal)	Alcohol Cannabis Stimulant Opioid Other	4	5.10	0.277
ARC (Social)	Alcohol Cannabis Stimulant Opioid Other	4	8.10	0.088
ARC (Sum)	One SOC Two SOC Three or More SOC “Whatever is available”	3	8.17	0.043*
ARC (Personal)	One SOC Two SOC Three or More SOC “Whatever is available”	3	8.65	0.034*
ARC (Social)	One SOC Two SOC Three or More SOC “Whatever is available”	3	5.67	0.129

*Note:* For significant findings, post hoc tests found lower recovery capital among the “whatever is available” group compared to the one SOC group.

### 3.2 Are there differences in RC across number of SOC categories?

The Kruskal-Wallis test comparing RC across number of SOC categories (one, two, three or more, “whatever is available”) indicated differences in RC across number of SOC categories (Table 2). Post hoc comparisons using Dunn's test with Bonferroni corrections indicated a significant difference between participants who selected one SOC and participants who selected “whatever is available,” with participants who selected “whatever is available” typically reporting lower RC than participants who selected one SOC (Appendix, Table 11). No other pairings were significantly different.

A Kruskal-Wallis test indicated a significant difference in personal RC across number of SOC categories (Table 2). Post hoc comparisons indicated that there was a significant difference between those who selected one SOC and those who selected “whatever is available,” with those who selected “whatever is available” reporting lower RC than those who selected one SOC (Appendix, Table 12). No other pairings were significant. A Kruskal-Wallis test to compare social RC across number of SOC categories indicated no significant differences (Table 2).

## 4 Discussion

This exploratory study examined the relationship between SOC and RC among adolescents attending a RHS. Findings indicated that adolescents who reported “whatever is available” as their SOC generally had lower RC than those who reported only one SOC. These results contribute to the adolescent recovery literature by (a) identifying differences in RC across number of SOC, (b) finding that RC did not differ across type of SOC, and (c) suggesting that assumptions underlying traditional single SOC questions may vary across developmental and recovery contexts.

Over a quarter of study participants (26.3%) selected preference of “whatever is available,” indicating that this preference is not an anomaly. The high prevalence of this suggests many adolescents have preferences for substances outside of seeking targeted effects from one or more substances. To our knowledge, no prior empirical studies have examined this use preference. There is a dearth of information on this use preference and a need for further examination to support recovery service planning.

Participants who selected preference for “whatever is available” reported lower personal RC than participants who selected preference for only one SOC. Additional investigation is needed to determine if this group is qualitatively different from the others. For example, they may have higher SUD severity or higher prevalence of other mental health disorders. No differences in social RC were observed across the number of SOC. Given the importance of social networks in recovery (e.g., Best et al., 2016; Jurinsky et al., 2023), this finding requires further exploration to understand why social RC did not differ across number of SOC while personal RC did. Additionally, this study did not find a relationship between the type of SOC and RC among adolescents attending a RHS. Future work may explore perceptions of different SOC and how stigma may impact perceived risk and outcomes for different SOC.

The current study has several limitations. First, the exploratory nature of the study warrants cautious interpretation of findings. Second, the sample of adolescents attending a RHS may not generalize to other adolescents in recovery. Further research should examine this relationship among different populations and in different recovery support settings. Third, missing data may have affected the reliability and validity of findings. However, there was not a systematic pattern of missingness, as determined by Little's MCAR test (Little, 1988). Fourth, there were not validated measures for SOC or RC for adolescents. Fifth, the response option “whatever is available” may hold different meanings to different individuals.

The study has several strengths. First, including use preferences of “whatever is available” makes an empirical contribution to an understudied area with possible practical usefulness. Second, recruiting adolescents in recovery advances our understanding of RC among this population, whom a growing body of research has identified as critical to informing developmentally appropriate supports (Finch et al., 2020; Hennessy et al., 2019; Jurinsky et al., 2023). Third, the sample of adolescents in recovery is the largest empirically explored to date. Fourth, the use of a nonparametric test accounted for non-normality of the data.

### 4.1 Directions for future research and practice implications

Further research is needed to examine SOC and RC. Researchers may explore this relationship across varying populations and with different RC measures. Additionally, future studies may benefit from qualitative and longitudinal methods. Researchers who ask participants about SOC may also consider allowing participants to select multiple substances and including an option for “whatever is available” to better capture use preferences. Future work may examine the utility of SOC measurement and compare preferences to use patterns. Recovery support services may study other ways to bolster RC, particularly personal RC, among individuals who prefer “whatever is available.”

## 5 Conclusions

The current study identified a relationship between SOC and RC. Participants who selected preference for “whatever is available” reported, on average, lower overall RC and lower personal RC than participants who selected preference for one SOC. These findings can help guide future research and practice to support positive recovery trajectories.

## Disclaimers

The authors have no disclaimers.

## Ethnical approval statement

The data used in this study were collected as part of the Systematic Evaluation of Adolescent Recovery Services (SEARS). The data collection protocol was reviewed by the Tufts University Institutional Review Board and determined to constitute non–human subjects research (IRB ID: STUDY00005418; determination date: October 29, 2024); therefore, formal IRB approval was not required.

## Declaration of generative AI in scientific writing

No Generative AI was used in the writing of this paper.

## CRediT authorship contribution statement

**Jules Boyle:** Writing – original draft, Formal analysis, Data curation, Conceptualization. **Jordan Jurinsky:** Writing – review & editing, Supervision, Resources, Project administration, Data curation, Conceptualization.

## Funding

This research did not receive any specific grant from funding agencies in the public, commercial, or not-for-profit sectors. This work was funded by salary support from 10.13039/100008090Tufts University.

## Declaration of competing interest

The authors declare that they have no known competing financial interests or personal relationships that could have appeared to influence the work reported in this paper.

## Data Availability

Research data are not shared.

## Appendix A Supplementary data

Supplementary material Supporting methodological information and additional analyses
