# Supplementary material for: Recovery capital and substance(s) of choice among adolescents: Exploratory analyses
Source: Addict Behav Rep. 2026 Oct 13;24:100759. doi: 10.1016/j.abrep.2026.100759 (PMC13683237; doi:10.1016/j.abrep.2026.100759)
Supplement: Supplementary file 1 [file mmc1.docx]

**Appendix**

**Table 3**

*Assessment of Recovery Capital (ARC) Modifications*

| **Item #** | **Original item** | **Modified item** |
| --- | --- | --- |
| 1.1 | I am currently completely sober and/or "clean" from drug use | I am currently completely sober |
| 1.3 | I have had no lapses or relapses regarding substance abuse | I have had no 'near things' about relapsing |
| 3.5 | I have no problems with transportation | I have no problems getting around |
| 4.1 | I am proud of the community I live in and feel a part of it | I am proud of the community I live in and feel part of it - sense of belonging |
| 9.4 | I look after my health | I look after my health and wellbeing |
| 9.5 | I meet all my obligations promptly (things you have made a commitment to do) | I meet all of my obligations promptly |

**Table 4**

*Frequencies for SOC Groups and Subgroups*

| Group | *n* | Percentage | |
| --- | --- | --- | --- |
|  |  | SOC Group | Total Sample |
| One SOC | 172 | - | - |
| Alcohol | 27 | 15.70% | 4.33% |
| Cannabis | 110 | 63.95% | 17.63% |
| Stimulant | 6 | 3.49% | 0.96% |
| Opioid | 8 | 4.65% | 1.28% |
| Other | 21 | 12.21% | 3.37% |
| Two SOC | 138 | - | - |
| Alcohol-Cannabis | 59 | 42.75% | 9.46% |
| Cannabis-Other | 39 | 28.26% | 6.25% |
| Alcohol-Other | 7 | 5.07% | 1.12% |
| Cannabis-Opioid | 7 | 5.07% | 1.12% |
| Opioid-Other | 6 | 4.35% | 0.96% |
| Other Combination | 20 | 14.49% | 3.21% |
| Three or More SOC | 150 | - | - |
| Alcohol-Cannabis-Other | 40 | 26.67% | 6.41% |
| Alcohol-Cannabis-Stimulant-Other | 16 | 10.67% | 2.56% |
| Cannabis-Opioid-Other | 16 | 10.67% | 2.56% |
| Cannabis-Stimulant-Other | 14 | 9.33% | 2.24% |
| Alcohol-Cannabis-Stimulant | 11 | 7.33% | 1.76% |
| Alcohol-Cannabis-Opioid | 7 | 4.67% | 1.12% |
| Alcohol-Cannabis-Opioid-Other | 7 | 4.67% | 1.12% |
| Cannabis-Multiple Other | 6 | 4.00% | 0.96% |
| Other Combination | 33 | 22.00% | 5.29% |
| “Whatever is available” | 164 | - | 26.3% |

*Note*. Combinations with *n* < 5 were included in a larger grouping, “Other Combination”.

**Table 5**

*Means, Medians, Standard Deviations, and IQRs for Type of SOC and Recovery Capital*

| Primary SOC | *n* | Mean | Median | SD | IQR |
| --- | --- | --- | --- | --- | --- |
| Alcohol | 27 | 41.7 | 46 | 9.7 | 9.0 |
| Cannabis | 110 | 38.9 | 40 | 7.8 | 12.5 |
| Stimulant | 6 | 36.5 | 39 | 8.3 | 13.5 |
| Opioid | 8 | 42.3 | 43 | 5.9 | 12.0 |
| Other | 21 | 35.8 | 38 | 14.39 | 16.0 |

*Note.* The information is among participants who selected preference of one SOC (*n* = 172)

**Table 6**

*Means, Medians, Standard Deviations, and IQRs for Type of SOC and Personal Recovery Capital*

| Primary SOC | *n* | Mean | Median | SD | IQR |
| --- | --- | --- | --- | --- | --- |
| Alcohol | 27 | 16.4 | 18.4 | 3.9 | 5.6 |
| Cannabis | 110 | 15.3 | 15.2 | 3.4 | 5.6 |
| Stimulant | 6 | 14.1 | 15.6 | 4.3 | 4.4 |
| Opioid | 8 | 16.2 | 16.4 | 3.4 | 6.0 |
| Other | 21 | 13.9 | 14.4 | 5.9 | 7.2 |

*Note.* The information is among participants who selected preference of one SOC (*n* = 172)

**Table 7**

*Means, Medians, Standard Deviations, and IQRs for Type of SOC and Social Recovery Capital*

| Primary SOC | *n* | Mean | Median | SD | IQR |
| --- | --- | --- | --- | --- | --- |
| Alcohol | 27 | 17.0 | 19.2 | 4.2 | 2.4 |
| Cannabis | 110 | 15.9 | 16.8 | 3.3 | 4.8 |
| Stimulant | 6 | 15.1 | 15.6 | 3.3 | 4.6 |
| Opioid | 8 | 17.6 | 18.0 | 1.7 | 1.4 |
| Other | 21 | 14.7 | 16.0 | 5.8 | 5.6 |

*Note.* The information is among participants who selected preference of one SOC (*n* = 172)

**Table 8**

*Means, Medians, Standard Deviations, and IQRs for Number of SOC Categories and Recovery Capital*

| SOC Number | *n* | Mean | Median | SD | IQR |
| --- | --- | --- | --- | --- | --- |
| One SOC | 172 | 39.0 | 41 | 9.2 | 13.3 |
| Two SOC | 138 | 38.8 | 40 | 7.8 | 10.0 |
| Three or More SOC | 150 | 37.5 | 39 | 8.9 | 12.8 |
| Whatever is Available | 164 | 36.7 | 39 | 9.1 | 11.3 |

**Table 9**

*Means, Medians, Standard Deviations, and IQRs for Number of SOC Categories and Personal Recovery Capital*

| SOC Number | *n* | Mean | Median | SD | IQR |
| --- | --- | --- | --- | --- | --- |
| One SOC | 172 | 15.3 | 16.0 | 3.9 | 5.6 |
| Two SOC | 138 | 15.2 | 15.2 | 3.5 | 5.6 |
| Three or More SOC | 150 | 14.5 | 15.2 | 4.1 | 5.4 |
| Whatever is Available | 164 | 14.3 | 14.4 | 3.8 | 5.6 |

**Table 10**

*Means, Medians, Standard Deviations, and IQRs for Number of SOC Categories and Social Recovery Capital*

| SOC Number | *n* | Mean | Median | SD | IQR |
| --- | --- | --- | --- | --- | --- |
| One SOC | 172 | 16.0 | 16.8 | 3.8 | 5.6 |
| Two SOC | 138 | 15.8 | 16.8 | 3.4 | 4.0 |
| Three or More SOC | 150 | 15.4 | 16.0 | 3.6 | 5.6 |
| Whatever is Available | 164 | 15.1 | 16.0 | 3.9 | 5.6 |

**Table 11**

*Pairwise Comparisons for Number of SOC Categories and Recovery Capital*

| Pairwise Comparison | Z-test statistic | *p* (Unadjusted) | *p* (Adjusted) |
| --- | --- | --- | --- |
| One SOC-Two SOC | 0.77 | .220 | 1.000 |
| One SOC-Three or More SOC | 1.82 | .034 | .205 |
| One SOC-“Whatever is available” | 2.68 | .004 | .022* |
| Two SOC-Three or More SOC | -0.98 | .164 | .982 |
| Two SOC-“Whatever is available” | 1.77 | .038 | .229 |
| Three or More SOC-“Whatever is available” | 0.79 | .215 | 1.000 |

*Note.* Pairwise comparisons were calculated using Dunn’s post hoc test with Bonferroni corrections. Adjusted p-values are multiplied by *m* (number of comparisons), *m* = 6

**Table 12**

*Pairwise Comparisons for Number of SOC Categories and Personal Recovery Capital*

| Pairwise Comparison | Z-test statistic | *p* (Unadjusted) | *p* (Adjusted) |
| --- | --- | --- | --- |
| One SOC-Two SOC | 0.47 | .319 | 1.000 |
| One SOC-Three or More SOC | 1.58 | .057 | .344 |
| One SOC-“Whatever is available” | 2.73 | .003 | .019* |
| Two SOC-Three or More SOC | -1.04 | .150 | .897 |
| Two SOC-“Whatever is available” | 2.11 | .017 | .104 |
| Three or More SOC-“Whatever is available” | 1.08 | .141 | .845 |

*Note.* Pairwise comparisons were calculated using Dunn’s post hoc test with Bonferroni corrections. Adjusted p-values are multiplied by *m* (number of comparisons), *m* = 6
